# A hierarchical Bayesian model for understanding the spatiotemporal dynamics of the intestinal epithelium

**DOI:** 10.1371/journal.pcbi.1005688

**Published:** 2017-07-28

**Authors:** Oliver J. Maclaren, Aimée Parker, Carmen Pin, Simon R. Carding, Alastair J. M. Watson, Alexander G. Fletcher, Helen M. Byrne, Philip K. Maini

**Affiliations:** 1 Department of Engineering Science, University of Auckland, Auckland, New Zealand; 2 Gut Health and Food Safety Research Programme, Institute of Food Research, Norwich, United Kingdom; 3 Norwich Medical School, University of East Anglia, Norwich, United Kingdom; 4 School of Mathematics and Statistics, University of Sheffield, Sheffield, United Kingdom; 5 Bateson Centre, University of Sheffield, Sheffield, United Kingdom; 6 Wolfson Centre for Mathematical Biology, Mathematical Institute, University of Oxford, Oxford, United Kingdom; University of California Irvine, UNITED STATES

## Abstract

Our work addresses two key challenges, one biological and one methodological. First, we aim to understand how proliferation and cell migration rates in the intestinal epithelium are related under healthy, damaged (Ara-C treated) and recovering conditions, and how these relations can be used to identify mechanisms of repair and regeneration. We analyse new data, presented in more detail in a companion paper, in which BrdU/IdU cell-labelling experiments were performed under these respective conditions. Second, in considering how to more rigorously process these data and interpret them using mathematical models, we use a probabilistic, hierarchical approach. This provides a best-practice approach for systematically modelling and understanding the uncertainties that can otherwise undermine the generation of reliable conclusions—uncertainties in experimental measurement and treatment, difficult-to-compare mathematical models of underlying mechanisms, and unknown or unobserved parameters. Both spatially discrete and continuous mechanistic models are considered and related via hierarchical conditional probability assumptions. We perform model checks on both in-sample and out-of-sample datasets and use them to show how to test possible model improvements and assess the robustness of our conclusions. We conclude, for the present set of experiments, that a primarily proliferation-driven model suffices to predict labelled cell dynamics over most time-scales.

## Author summary

The intestinal epithelium is an important model system for studying the dynamics and regulation of multicellular populations. It is characterised by rapid rates of self-renewal and repair; dysregulation of these processes is thought to explain, in part, why many tumours form in the intestinal and similar epithelial tissues. These features have led to a large amount of work on estimating cell kinetic parameters in the intestine. There remain, however, large gaps between the raw data collected, the interpretation of these experimental data, and mechanistic models that describe the underlying processes. Hierarchical statistical modelling provides a natural method with which to bridge these gaps, but has, to date, been underutilised in the study of intestinal tissue self-renewal. As we illustrate, this approach makes essential use of the distinction between ‘measurement’, ‘process’ and ‘parameter’ models, giving an explicit framework for combining experimental data and mechanistic modelling in the presence of multiple sources of uncertainty. We apply this approach to analyse experiments on healthy, damaged and recovering intestinal tissue, finding that observed data can be explained by a model in which cell movement is driven primarily by proliferation.

## Introduction

### Motivation

The intestinal epithelium provides crucial barrier, transport and homeostatic functions. These requirements lead it to undergo constant repair and regeneration, and dysfunctions can result in pathologies such as tumorigenesis [1–7]. Much work has been carried out on estimating the rate parameters in the intestine and other epithelia [1, 8–10]. However, attempts to interpret these experimental data using mechanistic modelling remain inconclusive [11–14], due to the lack of consistent and reproducible approaches for comparing models representing conjectured biological mechanisms, both to each other and to experimental data.

This challenge goes in both directions: using experimental data (taken to be ‘true’) to parameterise and test mathematical or computational formalisations of mechanistic theories, and using these models (taken to be ‘true’) to predict, interpret and question experimental results. Both experimental measurements and mathematical models are subject to uncertainty, and we hence need systematic ways of quantifying these uncertainties and attributing them to the appropriate sources. Furthermore, establishing a common approach for analysing experimental results, formulating mechanistic models and generating new predictions has many potential advantages for enabling interdisciplinary teams to communicate in a common language so that they may efficiently discover and follow promising directions as and when they arise.

### Approach

We address the above issues by developing a hierarchical Bayesian model for combining measurements, models and inference procedures, and applying it to a set of experiments targeting mechanisms of repair and regeneration in the intestinal epithelium.

While progress is now being made in tackling this challenge in other areas of biology (see, for example, [15–17]), to our knowledge the problem of intestinal epithelial dynamics has not yet been investigated using such an approach.

The experiments under investigation were performed by ourselves and are presented in more detail in [18]. The aim of these experiments was to determine how proliferation rates, tissue growth and cellular migration rates are related under healthy, damaged (Ara-C treated) and recovering conditions, and how these relations can be used to identify mechanisms of repair and regeneration.

A notable feature of the Bayesian approach to probabilistic modelling is that all sources of uncertainty are represented via probability distributions [19–21]. Adopting this perspective, we consider both observations and parameters to be random variables. Within a modelling context, uncertainty may be associated with (at least): parameters within a mechanistic model of a biological or physical process, the mechanistic model of the process itself and the measurements of the underlying process. This leads, initially, to the postulation of a full joint probability distribution for observable, unobservable/unobserved variables, parameters and data.

Another key feature of the Bayesian perspective is that it provides a natural way of decomposing such full joint models in a *hierarchical* manner, e.g. by separating out processes occurring on different scales and at different analysis stages. A given set of hierarchical assumptions corresponds to assuming a factorisation of the full joint distribution mentioned above, and gives a more interpretable and tractable starting point.

Our factorisation follows that described in [21–24]. This comprises: a ‘measurement model’, which defines the observable (sample) features to be considered reproducible and the precision with which they are reproducible (the measurement scale); an underlying ‘process’ model, which captures the key mechanistic hypotheses of spatiotemporal evolution, and a prior parameter model which defines a broad class of *a priori* acceptable parameter values.

In order to illustrate some of the modelling benefits of the hierarchical approach, we show how both discrete and continuous process models can be derived and related using the hierarchical perspective. We discuss the relationship between the conditional/hierarchical modelling and causal modelling literatures (see [25–27] for reviews) and illustrate the distinct roles of (Bayesian) predictive distributions vs. parameter distributions for model checking and the assessment of evidence, respectively [20, 28–31].

### Conclusions

Our hierarchical Bayesian model incorporates measurement, process and parameter models and facilitates separate checking of these components. This checking indicates, in the present application to the spatiotemporal dynamics of the intestinal epithelium, that much of the observed measurement variability can be adequately captured by a simple measurement model. Similarly we find that a relatively simple process model can account for the main spatiotemporal dynamics of interest; however, model checking also identifies a minor misfit in the process model appearing over long time-scales. This motivates possible model improvements: we consider the inclusion of additional finite-cell-size effects in the process model, derived from a discrete process model and a subsequent continuum approximation formulated in terms of conditional probability. This only gives a slightly better qualitative fit to experimental data, however. We instead find that the dominant sources of the long-time misfits are probably due to some other factors—most likely relatively slow, time-varying proliferation rates (e.g. due to circadian rhythms). In summary, a primarily proliferation-driven model appears adequate for predictions over moderate time-scales.

## Materials and methods

### Experimental treatments and data processing

#### Homeostasis mouse model

To estimate intestinal epithelial cell proliferation and migration rates under normal, homeostatic conditions in healthy mice, we used standard methods of proliferative cell labelling and tracing [1, 8–10, 32–34] (see also [18] for full details). Actively proliferating cells in the intestinal crypts were labelled by single injection of the thymine analogue 5-bromo-2-deoxyuridine (BrdU) and labelled cells detected by immunostaining of intestinal sections collected from different individuals over time. Migration of labelled cells traced from the base of crypts to villus tips was monitored over the course of 96 hours (5760 min). At least 30 such strips were analysed per mouse. The figures presented in [18] reveal that these strips were independent and obtained from one-cell thick sections. All strips in which the base of the crypt and the tip of the villus were clearly observed were considered. All sides of the crypt that were visible and connected to entire villi were analysed. A typical image from those analysed in [18] is shown in Fig D in S1 Supplementary information.

#### Blocked-proliferation mouse model

To assess the effects of proliferation inhibition on crypt/villus migration, migrating and proliferating epithelial cells were monitored by double labelling with two thymine analogues (BrdU and IdU), administered sequentially a number of hours apart and subsequently distinguished by specific immunostaining in longitudinal sections of small intestine. Following initial IdU labelling of proliferating cells at t = −17h (−1020 min, relative to Ara-C treatment), mice were then treated with cytosine arabinoside (Ara-C) at 250 mg/kg body weight, a dose reported to inhibit proliferation without causing major crypt cell atrophy (see [18] and references therein for details). Tissues were collected over 24 hours, with BrdU administered one hour prior to collection to check for residual proliferation. Successful inhibition of proliferation following treatment with Ara-C was confirmed by an absence of BrdU (S-phase) and phospho-Histone H3 (pH3) staining (M-phase) in longitudinal sections of small intestine (again, see [18] for full details).

#### Recovering-proliferation mouse model

The above Ara-C treatment effect was observed to last for at least 10h (600 min). Cell proliferation returned to near normal levels in samples obtained 18h (1080 min) post-Ara-C treatment. We hence considered samples collected at least 10h post-Ara-C treatment as corresponding to ‘recovering-proliferation’ conditions.

#### Data processing: Reference grid and key observable features

To relate experimental measurements to the mechanistic models discussed below we specified a reference grid and defined the key features of the data relative to this grid. These key features established an ideal ‘underlying population’ from which samples were considered to be drawn. This also allowed us to construct our hierarchical model in a (data-to-parameter) manner, starting from a measurement model.

**Fig 1:**
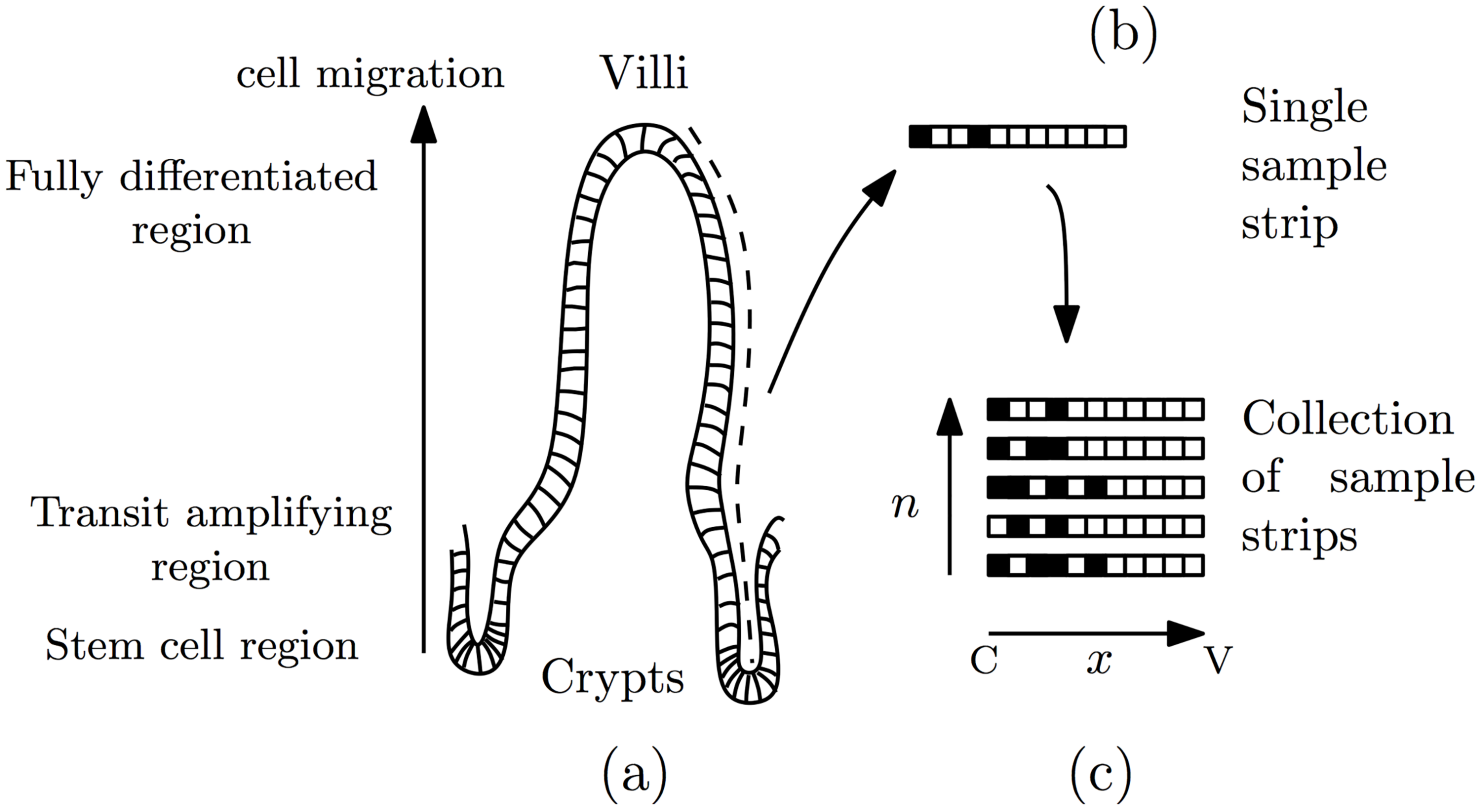
The (a) intestinal epithelium, (b) individual measurements as strips of cells and (c) collection of strips, where ‘C’ and ‘V’ indicated ‘crypt’ and ‘villus’ respectively.

With reference to Fig 1, we viewed the data as a collection of one-dimensional ‘strips’ of cells. The strips extended from the base of the crypt to the tip of the villus, along the so-called ‘crypt-villus’ axis. This corresponds to how strips were collected experimentally, but does not account for possible biases due to ‘angled’ sampling [35, 36]. Each measurement was given a spatial cell location index *i* and a time label *t*. The location index was measured in numbers of cells along the crypt-villus axis, starting from the crypt base, and hence defined a discrete one-dimensional grid.

When notationally convenient, the labels *i* and *t* were combined in a two-dimensional grid of space-time points via the single index parameter *s* ≔ (*i*, *t*). A ‘typical’ reference crypt-villus unit was characterised by the two vectors (**L**, **n**), where **L** is the vector of underlying labelled fractions (i.e. occupancy probabilities) at each grid point and **n** is the vector of number of samples at each grid point. This defines a useful reduction of the system from two spatial dimensions to one.

We assumed that each strip was independent of the others as, in general, strips were taken from different crypt-villus units and/or animals after ‘identical preparation’. Thus we did not ever directly possess, for example, measurements of a particular crypt with dimensions given in terms of a certain number of strips. We note, however, that the dynamics of strips in a given crypt may be affected by those in the same crypt. We did not consider this additional complexity in the present work, and so this complication should be kept in mind when interpreting the results.

### Mathematical model

Our hierarchical probability model was constructed on the basis of conditional probability assumptions. These allowed us to factor out a measurement model, a mechanistic model and a parameter model.

#### Overall hierarchical structure

Our model structure consisted of a full joint distribution, conditioned on a given experimental treatment *E* and known sample size vector **n**, decomposed according to

$$\begin{array}{c} p(y,L,k|n,E)=p(y|L,n)p(L|k)p(k|E), \end{array}$$ (1)

where **k** are the cellular proliferation rates (these are discussed below). This hierarchical factorisation corresponds to the assumption of conditional independence between the various levels, i.e. *p*(**y**|**L**, **k**, **n**, *E*) = *p*(**y**|**L**, **n**), *p*(**L**|**k**, **n**, *E*) = *p*(**L**|**k**) and *p*(**k**|**n**, *E*) = *p*(**k**|*E*). The first term, *p*(**y**|**L**, **n**) is the *measurement model*; the second term *p*(**L**|**k**) is the underlying *process model*, and the last term *p*(**k**|*E*) is a *prior parameter model*. These are discussed in turn below.

Notably, a ‘causal’ (structural invariance) assumption [25–27, 37–42] is made by assuming that the experimental treatment condition affects the process parameters **k** but not the structure of the measurement or process models. In particular, while the experimental treatment ultimately affects **y**, in our model it does so *via* its effect on **k** and **k**’s subsequent effect on **L**. This leads to the conditional independence structure mentioned. In terms of so-called directed acyclic graphs (DAGs), used in the causal modelling literature referenced above, we assumed

E→k→L→y. (2)

Another way of stating this is that knowledge of **L** (and **n**) is sufficient to determine **y** regardless of how **L** was brought about, but that to know **L** we (ultimately) have to know which experiment was carried out. Note that in general we suppressed, in our notation, the explicit conditioning on sample size **n**, since it was taken to be fixed and known, as well as the conditioning on *E* (keeping in mind that it was assumed to only affect **k**).

The assumptions underlying the above factorisation could be checked to some extent. This relied on a distinction between working ‘within’ the model—e.g. parameter estimation assuming the model and factorisation is valid—and working ‘outside’ the model, e.g. checking the validity of the model structural assumptions themselves [20, 30, 31]. This distinction is made in the Results section.

Implicit in the model derivations, discussed below, we used a *deterministic expression of conservation of probability* for the process model, as is typical for such equations [43]. It sufficed for the presentation here to simply replace all functional dependencies on the process variable above with a dependence on the process parameters [21].

We provide further discussion of our approach to interpretation of statistical evidence in the Supplementary Information.

#### Bayesian framework for predictions and incorporating information from observations

The overall model of the previous section defined our initial ‘generative’ probabilistic model, prior to explicitly incorporating information from our experimental data. This enabled samples to be drawn from both prior predictive and prior parameter models, in the usual way (see e.g. [20, 44] and the Computational methods section below). In particular, the prior predictive distribution was used in its usual form

$$\begin{array}{c} y∼p(y)=\int p(y|f(k))p(k)dk \end{array}$$ (3)

which incorporates the aforementioned deterministic link between a given sample of process parameters and the output process variable, **L** = *f*(**k**). Note that here ∼ denotes ‘distributed as’, or more relevantly, ‘samples drawn according to’.

To incorporate new data **y**_**0**_ we updated the parameters of the model, hence passing to a ‘posterior predictive’ model [20]

$$\begin{array}{c} y|y_{0}∼p\left(y|y_{0}\right)=\int p\left(y|f\left(k\right)\right)p\left(k|y_{0}\right)dk \end{array}$$ (4)

where we used the conditional probability closure assumption *p*(**y**|*f*(**k**), **y**_**0**_) = *p*(**y**|*f*(**k**)). This closure assumption can be interpreted as maintaining our same mechanistic model despite new observations.

#### Measurement model

The measurement model *p*(**y**|**L**, **n**) component was taken to be a binomial distribution B of the form

$$\begin{array}{c} p\left(y|L,n\right)=\underset{s=0}{\overset{S-1}{\Pi }}B\left(n_{s},L_{s}\right). \end{array}$$ (5)

This related our ‘raw’ observable **y**, the vector of counts of labelled cells at each grid point, to ‘ideal characteristics’ of comparison (**L**, **n**). We took the measurement component to be independent of the experimental treatment *E*, i.e. treatment was assumed to affect the underlying *process parameters* only (see ‘Overall hierarchical structure’ section). The measurement component *p*(**y**|**L**, **n**) then defined a likelihood function L for this measurement model,

$$\begin{array}{c} L\left(L;y,n\right)=\underset{s=0}{\overset{S-1}{\Pi }}L_{s}^{y_{s}}{\left(1-L_{s}\right)}^{n_{s}-y_{s}}\propto p\left(y|L,n\right)=\underset{s=0}{\overset{S-1}{\Pi }}B\left(n_{s},L_{s}\right). \end{array}$$ (6)

When interpreting model misfit based on residuals, we applied the usual normal approximation to the binomial distribution. In that case, denoting the set of all measured labelled fractions through the (useful, but slightly non-standard) notation **y**/**n** ≔ (*y*_1_/*n*_1_, …, *y*_*S*_/*n*_*S*_), we have

$$\begin{array}{c} L\left(L;n,\frac{y}{n}\right)=p\left(\frac{y}{n}|L,n\right)=\underset{s=0}{\overset{S-1}{\Pi }}\frac{1}{\sigma_{s}\sqrt{2\pi }}\exp\left(-\frac{{\left(\frac{y_{s}}{n_{s}}-L_{s}\right)}^{2}}{2\sigma_{s}^{2}}\right) \end{array}$$ (7)

where the standard deviations are given by $\sigma_{s}=\sqrt{\frac{L_{s}\left(1-L_{s}\right)}{n_{s}}}$.

#### Process models: Spatially discrete model

We developed our process model at different levels of resolution. First, we considered a spatially discrete probabilistic model at the level of our measurement grid defined above. Second, we considered two spatially continuous approximations: one model excluding explicit cell-scale effects and one including them. Here we first consider the discrete model.

Our basic ‘process’ model described the evolution of the occupancy probabilities (population labelled fractions) at the scale of the measurement grid. This was derived as follows. With reference to Fig 1, we considered a collection of one-dimensional ‘strips’ of cells. We used *l*_*i*_ ∈ {0, 1} as an indicator variable denoting the occupancy status of site *i* of a given strip. The full state of this strip was given by the vector **l** = (*l*_0_, *l*_1_, …*l*_*S* − 1_). We then sought a description of the probabilistic dynamics in terms of a discrete-time Markov chain for the probability distribution of the full state *p*(**l**, *t*) following standard arguments [43, 45].

We began from an explicit joint distribution for the full state and then reduced it to a description in terms of the set of ‘single-site’ probability distributions *p*(*l*_*i*_, *t*) for each site *i*. This derivation was aided by adopting an explicit notation: the probabilities of occupancy and vacancy at site *i* at time *t* were denoted by *p*(*l*_*i*_(*t*) = 1) and *p*(*l*_*i*_(*t*) = 0) respectively. Since *p*(*l*_*i*_(*t*) = 1) + *p*(*l*_*i*_(*t*) = 0) = 1 we only needed to consider the probability of occupancy to fully characterise the distribution *p*(*l*_*i*_(*t*)).

The equation of evolution for this probability was derived by considering conservation of probability in terms of probability fluxes in and out, giving, to first order in Δ*t*,

$$\begin{array}{c} p(l_{i}\left(t+\Delta t\right)=1)-p(l_{i}\left(t\right)=1)=\Delta t\sum_{j=0}^{i-1}k_{j}[p\left(l_{i-1}\left(t\right)=1,l_{i}\left(t\right)=0\right)-p\left(l_{i-1}\left(t\right)=0,l_{i}\left(t\right)=1\right)]. \end{array}$$ (8)

The first term on the right represents a net ‘influx of occupancy probability’ due to a single division event at site *j* < *i*, each division event having a probability *k*_*j*_ Δ*t*. This flux means the value of the state variable *l*_*i*_(*t*) = 0 could be replaced, at the next time step, by the value of *l*_*i* − 1_(*t*) = 1. Similarly the second term represents a net ‘outflux of occupancy probability’ due to a division event at site *j* < *i*. Partitioning on the events *l*_*i* − 1_(*t*) = 0 and *l*_*i*_(*t*), we obtain

$$\begin{array}{c} p\left(l_{i}\left(t+\Delta t\right)=1\right)-p\left(l_{i}\left(t\right)=1\right)=\Delta t\sum_{j=0}^{i-1}k_{j}[p\left(l_{i-1}\left(t\right)=1\right)-p\left(l_{i}\left(t\right)=1\right)]. \end{array}$$ (9)

#### Process models: Underlying continuous model and zeroth-order approximation

To aid model interpretation and model cross comparisons we derived a continuous approximation to the occupancy probability, *L*(*x*, *t*). This gave a further idealisation of the ‘underlying population’ from which we envisaged the strips were sampled. This smoothness assumption, while not strictly necessary, meant some model properties could be interpreted in terms of local derivatives; it also reduced arbitrary dependence on discrete grid features, aiding future comparisons with off-lattice and/or continuum models [44].

As detailed in the Supplementary Information, *L*(*x*, *t*) satisfies the equation

$$\begin{array}{c} \frac{\partial L(x,t)}{\partial t}+v\left(x\right)\frac{\partial L(x,t)}{\partial x}=0 \end{array}$$ (10)

with

$$\begin{array}{c} v\left(x\right)=\int_{0}^{x}k\left(x^{\prime }\right)dx^{\prime }, \end{array}$$ (11)

where *k*(*x*) denotes the (net) proliferation rate.

#### Process models: Underlying continuous model and higher-order spatial effects

Our ‘zeroth-order’ continuous approximation above was obtained by neglecting all higher-order terms in Δ*x*. We anticipate that a more accurate continuum approximation may be obtained by retaining higher-order spatial derivatives and hence finite-cell-size effects. This gives rise to a Fokker-Planck equation containing a diffusion term [43]:

$$\begin{array}{c} \frac{\partial L(x,t)}{\partial t}+v\left(x\right)\frac{\partial L(x,t)}{\partial x}=D\left(x\right)\frac{\partial^{2}L\left(x,t\right)}{\partial x^{2}} \end{array}$$ (12)

where *D*(*x*) = (1/2)Δ*xv*(*x*). Similar equations have been derived before, based on continuous approximations to discrete master equations [46–49]. Retaining the second spatial derivative hence amounts to accounting for spatial effects due to finite cell sizes. We evaluated our original ‘zeroth-order’ (advection) model against our data, and also examined the extent to which higher-order spatial terms such as those considered above could account for any misfits.

#### Definition of priors

Since we adopted a Bayesian perspective in this work we required a parameter prior model that could express additional modelling assumptions [20]. Essentially, this is an empirical Bayesian approach as we used an empirical correlation matrix [50].

Candidate proliferation profiles, varying with cell locations, were represented as realisations from a prior given in terms of a discretised random field (a random vector) **k** of length *m* = 5, modelled as a multivariate Gaussian N(μ,C) with joint distribution

$$\begin{array}{c} p\left(k\right)=\frac{1}{{\left(2\pi \right)}^{m}\sqrt{\text{det}(C)}}\exp\left(-{\left(k-\mu \right)}^{T}C^{-1}\left(k-\mu \right)/2\right) \end{array}$$ (13)

characterised by its mean vector ***μ*** and covariance matrix **C**. This parameter prior constrained the variability of the spatially varying parameter field *a priori* to help avoid unphysical solutions.

The covariance matrix was first decomposed into a standard deviation matrix given by the outer (tensor) product of the standard deviation vector for each variable, **S** = ***σσ***^*T*^, and correlation matrix **R**. These multiply element-wise to give *C*_*ij*_ = *S*_*ij*_ *R*_*ij*_ (no summation). We then adopted the common, equivalent, representation **C** = **DRD** where **D** is a diagonal matrix with diagonal entries *D*_*ii*_ = *σ*_*i*_.

We took the correlation matrix **R** to have the squared-exponential (Gaussian) correlation function $k\left(i,j\right)=\exp\left(\frac{{\left(i-j\right)}^{2}}{2l_{c}^{2}}\right)$, where *l*_*c*_ is a parameter controlling the characteristic length-scale of the correlations in terms of number of indices of **k**. This characteristic length scale gives the number of **k** indices over which the correlation function decays to 1/*e*. This allowed us to control the ‘smoothness’ of the realisations from the **k** prior, in the sense that as *l*_*c*_ is increased the values **k**_*i*_ and **k**_*j*_ tend to be more similar.

The matrix **R** was generated by evaluating this correlation function at discrete locations along the crypt-villus axis. This discretisation was chosen to be coarser than the measurement grid and gave a variation somewhat similar to compartment-style regions of proliferation activity. This corresponded to assuming that the cell-type and associated proliferation rates varied on a coarser scale than individual cells, and was thus somewhat similar to a compartment-style assumption [51, 52], though the resulting proliferation rate function is defined for all values of the finer, individual-cell scale *x*. The parameter *l*_*c*_ could also be interpreted as a ‘parameter correlation length’ for the proliferation rates, a measure of the number of parameters—or number of ‘compartments’—over which the correlations decay. We considered correlation lengths of 1–2 parameters.

We found it most informative to visualise realisations of the whole function from the resulting prior rather than simply give the individual parameters/matrices separately ([21] discusses this visualisation approach to priors in more detail). These are hence discussed and displayed in more detail in the Results section below.

#### Implementation of MCMC sampling and Bayesian updating

We used the (open source) Python package emcee (http://dan.iel.fm/emcee/) to perform Markov Chain Monte Carlo (MCMC) [53] to obtain samples from the posterior distributions. Given samples from the resulting prior and posterior parameter distributions, respectively, prior and posterior predictive distributions were obtained by forward simulation of the process model described below. We note that each candidate proliferation rate vector **k** is connected to the measurements **y** via the latent vector **L**; since this step is deterministic, however, no additional sampling steps were required for the process model component.

#### Solution of differential equations

For the results in all sections other than the final results section in which we include higher-order spatial effects, we solved the differential equation model using the *PyCLAW* [54, 55] Python interface to the *CLAWPACK* [56] set of solvers for hyperbolic PDEs. We adapted a Riemann solver for the colour equation available from the Riemann solver repository (https://github.com/clawpack/riemann). For testing the inclusion of higher-order spatial effects (thus changing the class of our equations from hyperbolic to parabolic) we used the Python finite-volume solver *FiPy* [57].

#### Data and source code availability

Our code is available in the form of a Jupyter Notebook (http://ipython.org/notebook.html) in the Supplementary Information. We ran these using the Anaconda distribution of Python (https://store.continuum.io/cshop/anaconda) which is a (free) distribution bundling a number of scientific Python tools. Any additional Python packages and instructions which may be required are listed at the beginning of our Jupyter Notebook.

## Results

### Parameter inference under homeostatic (healthy) conditions

**Fig 2:**
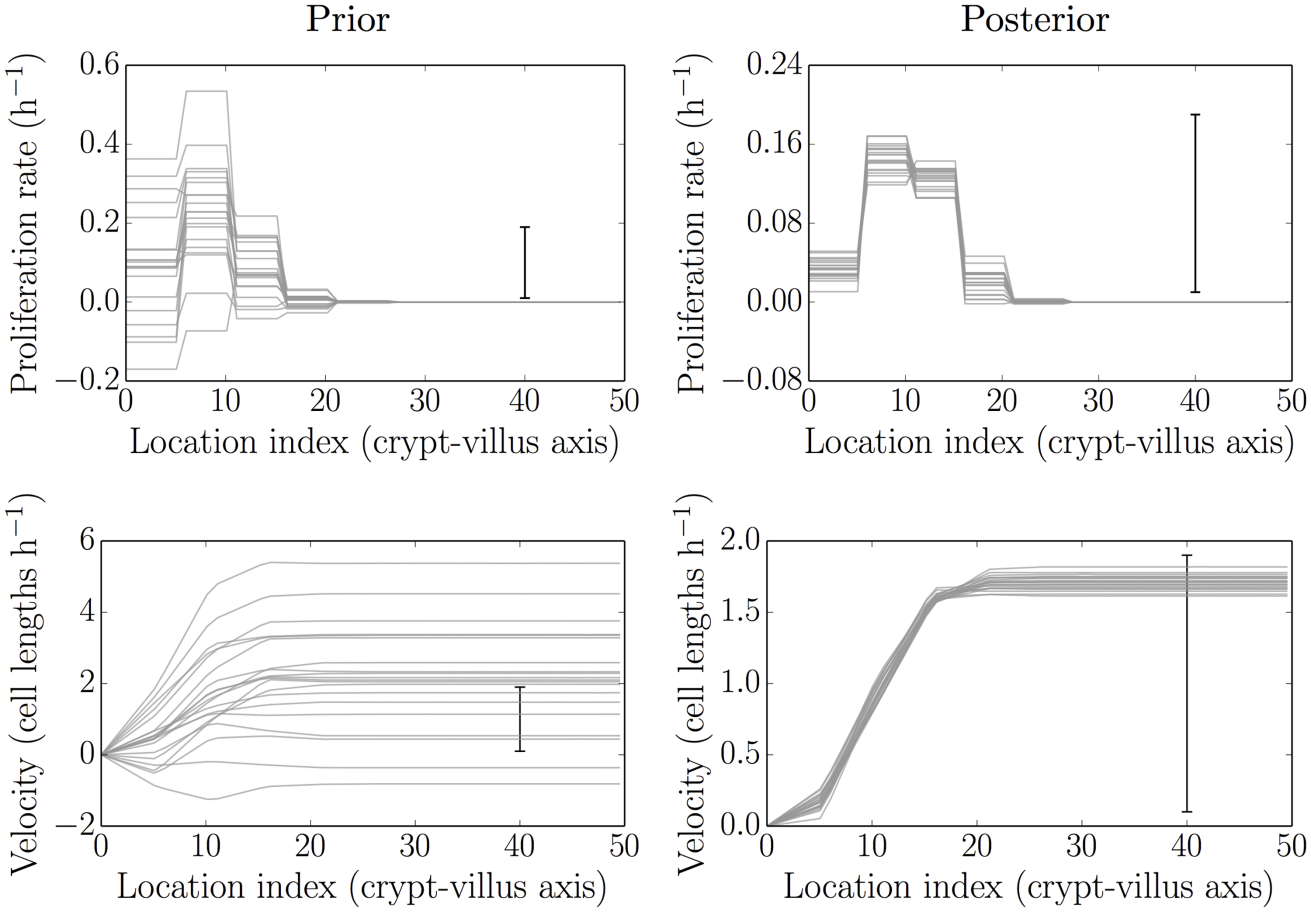
Simulated realisations from the prior (left) and posterior (right) distributions for proliferation profiles (top) and velocities (bottom). After data are obtained the posterior distributions are much more tightly-constrained, and are picking out biologically plausible results (see main text).

Fig 2 illustrates the process of updating from realisations of the prior distributions of the proliferation and velocity fields to realisations of their posterior (post-data) distributions. As discussed in the Materials and methods section above, these are generated by an underlying piecewise-constant Gaussian random field of proliferation rates, **k**. This has length *m* = 5, and defines an assignment of the cell indices into biologically-motivated regions of proliferation activity.

The left-hand side of the figure shows simulations from the prior distribution for proliferation field (top) and realisations from the induced distribution for the velocity field (bottom), respectively. The right-hand side shows the corresponding simulations after the prior parameter distribution has been updated to a posterior parameter distribution. The prior-to-posterior parameter estimation was carried out using the MCMC sampling approach described above with *t* = 120 min (2 h) as an initial condition and *t* = 360 min (6 h) and 600 min (10 h) as given data. The initial condition for the underlying labelled fraction (occupancy probability) was determined by fitting a smoothing spline to the data. The prior distribution for the proliferation field shown in Fig 2 incorporated a weak mean trend in net proliferation rates, rising from the crypt base to the mid-crypt before falling exponentially to zero over the last few parameter regions post-crypt end, and a parameter correlation length of 1. These assumptions can be relaxed/varied with little effect, though typically a non-zero parameter correlation length and a shut-off in proliferation after the crypt end produce more stable (well-identified) estimates. Additional visualisations of the parameter inferences are provided in Fig A-C in S1 Supplementary information.

### Parameter inference for blocked proliferation conditions

**Fig 3:**
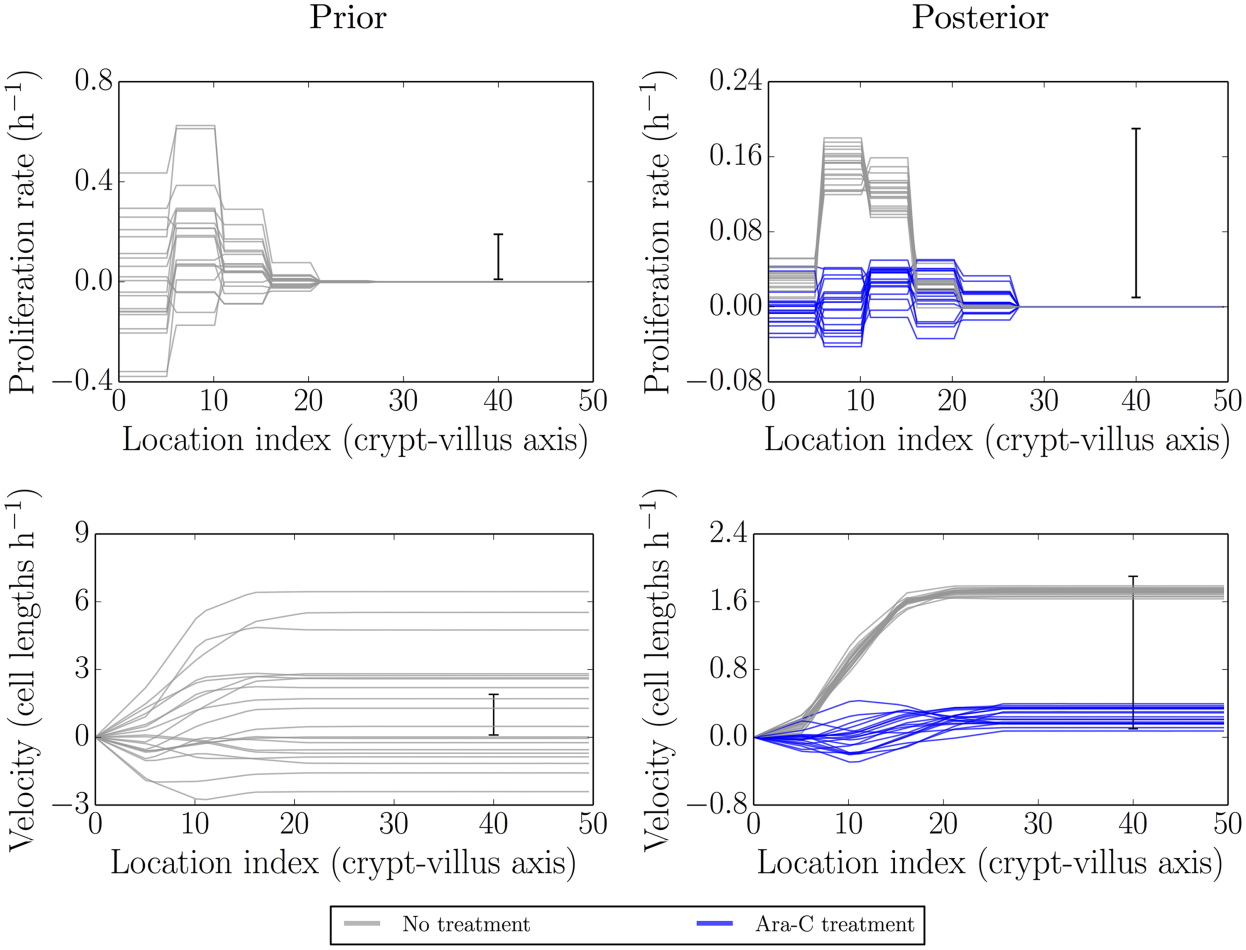
Simulated realisations from the prior (left) and posterior (right) distributions for proliferation profiles (top) and velocities (bottom) under Ara-C treatment (blue) as compared to no treatment (grey). The velocities are reduced to near zero, as are the proliferation rates, though the latter are noisier.

Fig 3 is the same as Fig 2 described in the previous section, but this time under treatment by Ara-C. Results from the baseline case are shown in grey, while those from Ara-C treatment are shown in blue. Here 1140 min (19 h post IdU labelling, 2 h post Ara-C treatment) was used as the initial condition and 1500 min (25 h post IdU labelling, 8 h post Ara-C treatment) used for fitting. The intermediate time 1260 min (21 h post IdU labelling, 4 h post Ara-C treatment) and later time 1620 min (27 h post IdU labelling, 10 h post Ara-C treatment) were used as out-of-sample comparisons (see later).

As can be seen, there is clear inhibition of proliferation and an even clearer effect on the cell migration (growth) velocity. The greater variability in the underlying parameter results compared to the baseline case may indicate, for example, greater parameter underdetermination and/or inconsistency of the model. This is not surprising as we expect all proliferation parameters to be reduced to similar (low) values and hence to become less distinguishable.

To add additional stability to the results we can attempt to reduce underdetermination in the parameters by increasing the parameter correlation length and inducing an effectively more ‘lumped’ representation of the parameter field (since values tend to stick together more). Doing this removed the more extreme negative net proliferation in the posterior profile, however it still allowed for small amounts of negative net proliferation/velocity (the available Jupyter notebook can be used to explore various prior assumptions).

Again, the need to introduce more stability is likely due to some combination of the limitations of resolution, a consequence of trying to fit the data too closely, or an indication of model inadequacies. In particular, under inhibited-proliferation conditions the effective number of parameters would be expected to be reduced. When fitting the full model, with largely independent parameters for each region, it is to be expected that some additional regularisation would be required for greater stability.

### Parameter inference for recovering proliferation conditions

Ara-C is metabolised between 10–12 h post-treatment. The two times considered here, 1620 min and 2520 min, correspond to 10 h and 25 h post Ara-C treatment, respectively, i.e to the end of the effect and after the resumption of proliferation. Hence, to check for the recovery of proliferation, we fitted the model using 1620 min as the initial condition and 2520 min as the final time.

**Fig 4:**
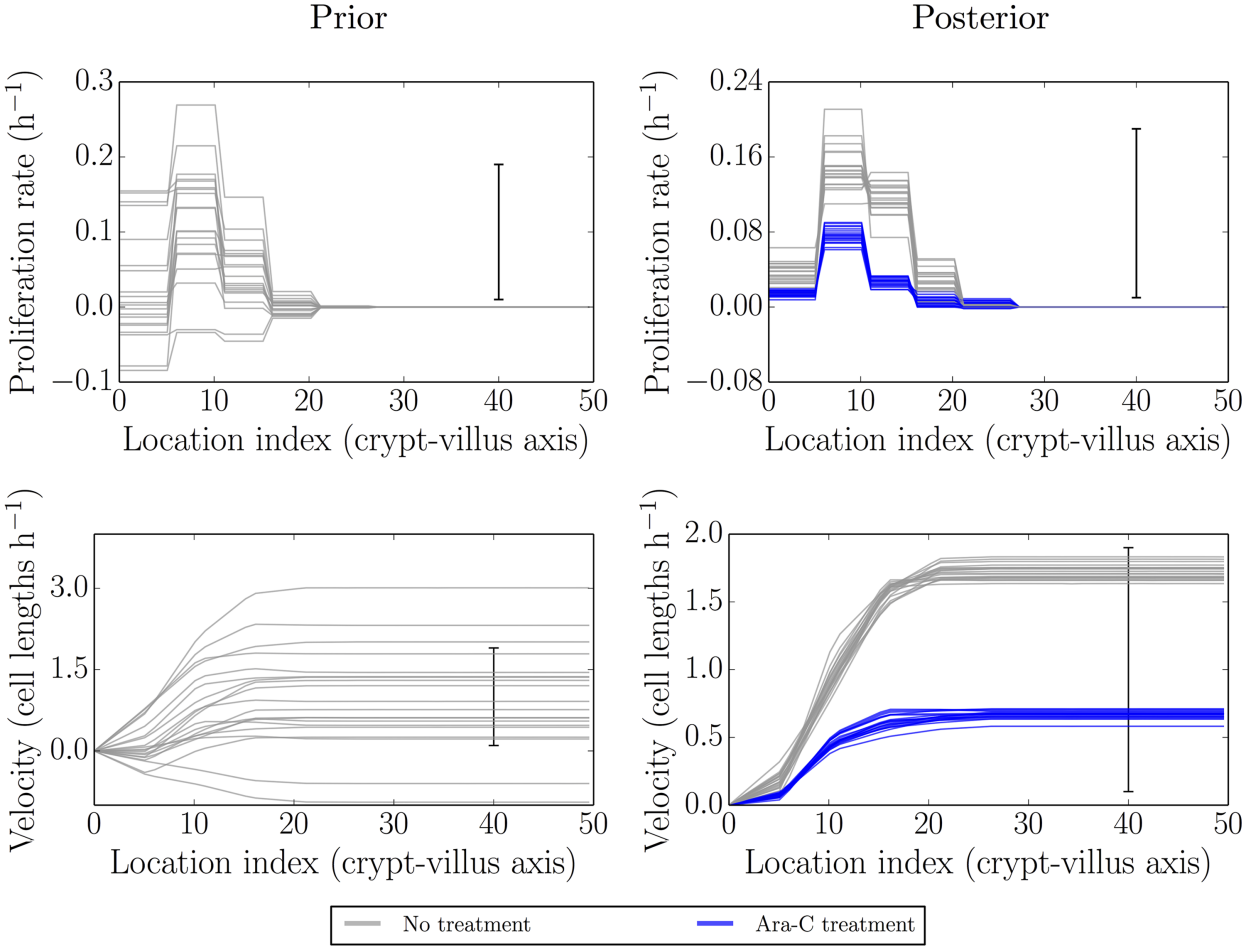
Simulated realisations from the prior (left) and posterior (right) distributions for proliferation profiles (top) and velocities (bottom) after recovery from Ara-C treatment (blue) as compared to no treatment (grey). The velocities and proliferation rates show partial recovery towards healthy conditions.

Fig 4 is the same as Figs 2 and 3 described in the previous sections, but this time after/during recovering from treatment by Ara-C. The previous results from the baseline case are shown in grey, while the new results following recovery from Ara-C treatment are shown in blue. Here 1620 min (27 h post IdU labelling, 10 h post Ara-C treatment) was used as the initial condition and 2520 min (42 h post IdU labelling, 25 h post Ara-C treatment) used for fitting. We did not make additional out-of-sample comparisons in this case, though in-sample posterior predictive checks were still carried out (see later).

Here, as expected, the proliferation and velocity profiles indicate that proliferation has resumed. The rates of proliferation appear to be lower than under fully healthy conditions, however, perhaps due to incomplete recovery (the initial condition being right at the beginning of the recovery period). The timing of the recovery of proliferation and the well-identified proliferation and velocity profiles inferred give no indication that any other mechanism is required to account for these data, however.

### Predictive checks under homeostatic (healthy) conditions

**Fig 5:**
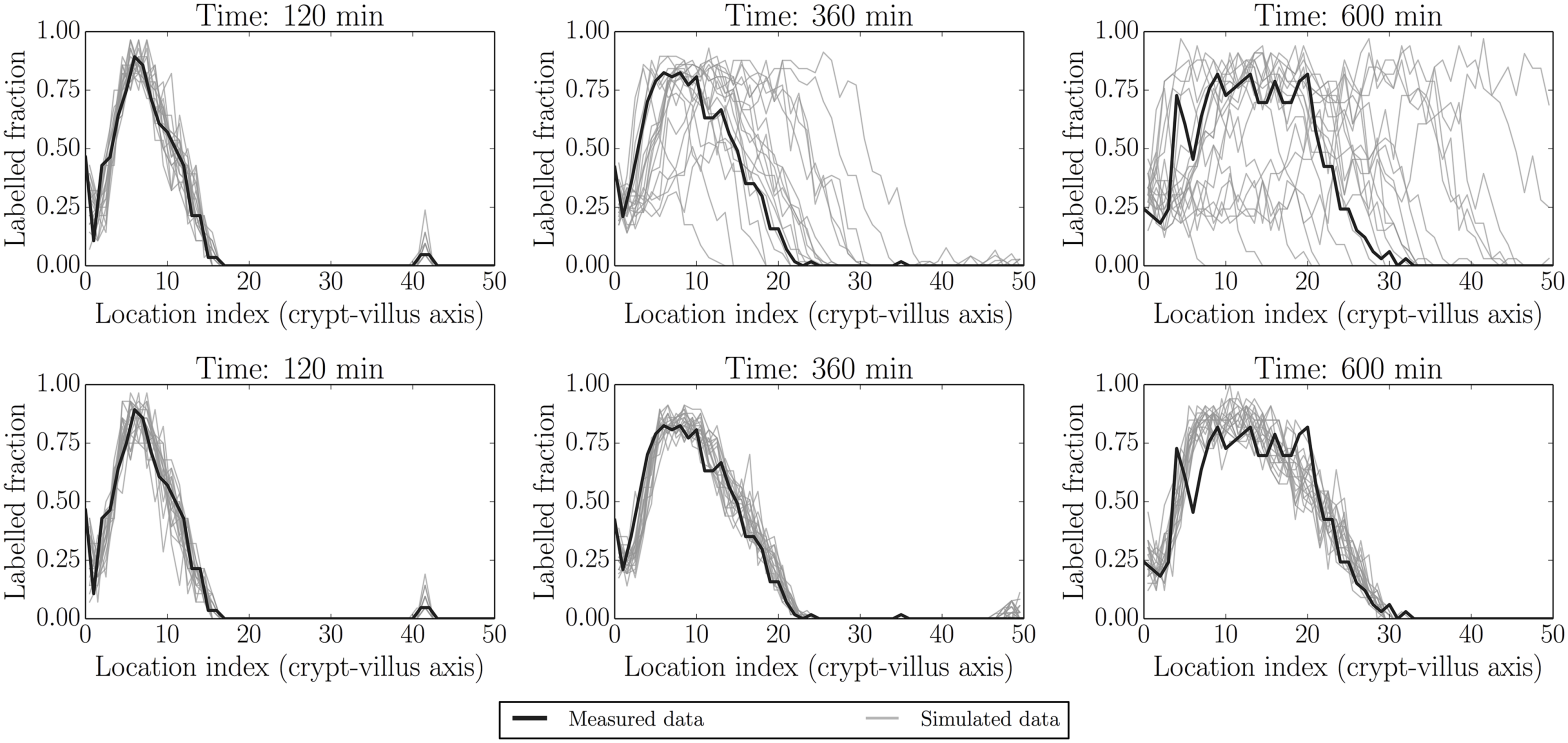
Simulated realisations from prior (top) and posterior (bottom) predictive distributions (grey) for label data at fitted times (120 min, 360 min and 600 min i.e. 2 h, 6 h and 10 h). Actual data are indicated by black lines. Again the posterior distributions are much more constrained than the prior distributions, representing the gain in information from collecting (and fitting to) experimental data. The first profile in each panel is held as a constant initial condition in this example.

Fig 5 illustrates simulations from the predictive distributions corresponding to the prior and posterior parameter distributions of Fig 2. This enables a first self-consistency check—i.e. can the model re-simulate data similar to that to which it was fitted [20, 58]? If this is the case then we can (provisionally) trust the parameter estimates in the previous figure; if not, then the parameter estimates would be unreliable, no matter how well-determined they seem. In our case the model appears to adequately replicate the data used for fitting.

**Fig 6:**
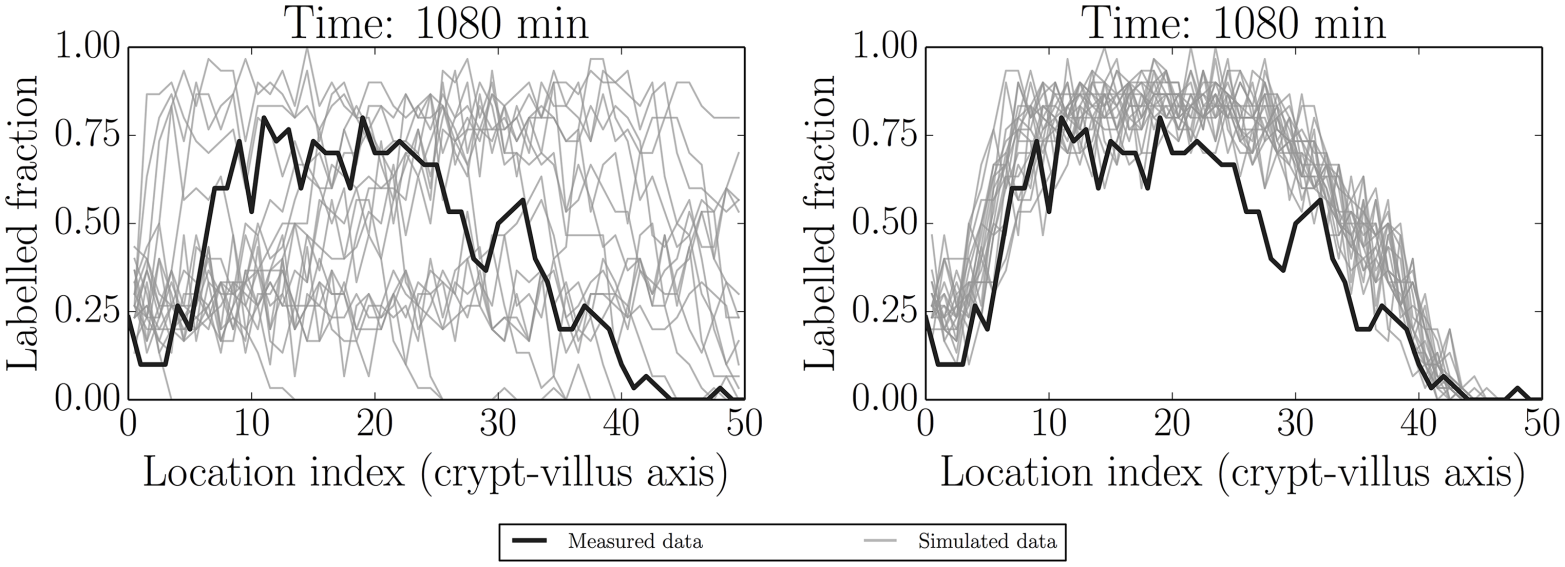
Simulated realisations from prior (left) and posterior (right) predictive distributions (grey) for label data at the unfitted (out-of-sample) time 1080 min (18 h). Actual data are indicated by black lines. The model appears to give reasonable predictions capturing the key features, but there is also some misfit to be explored further.

**Fig 7:**
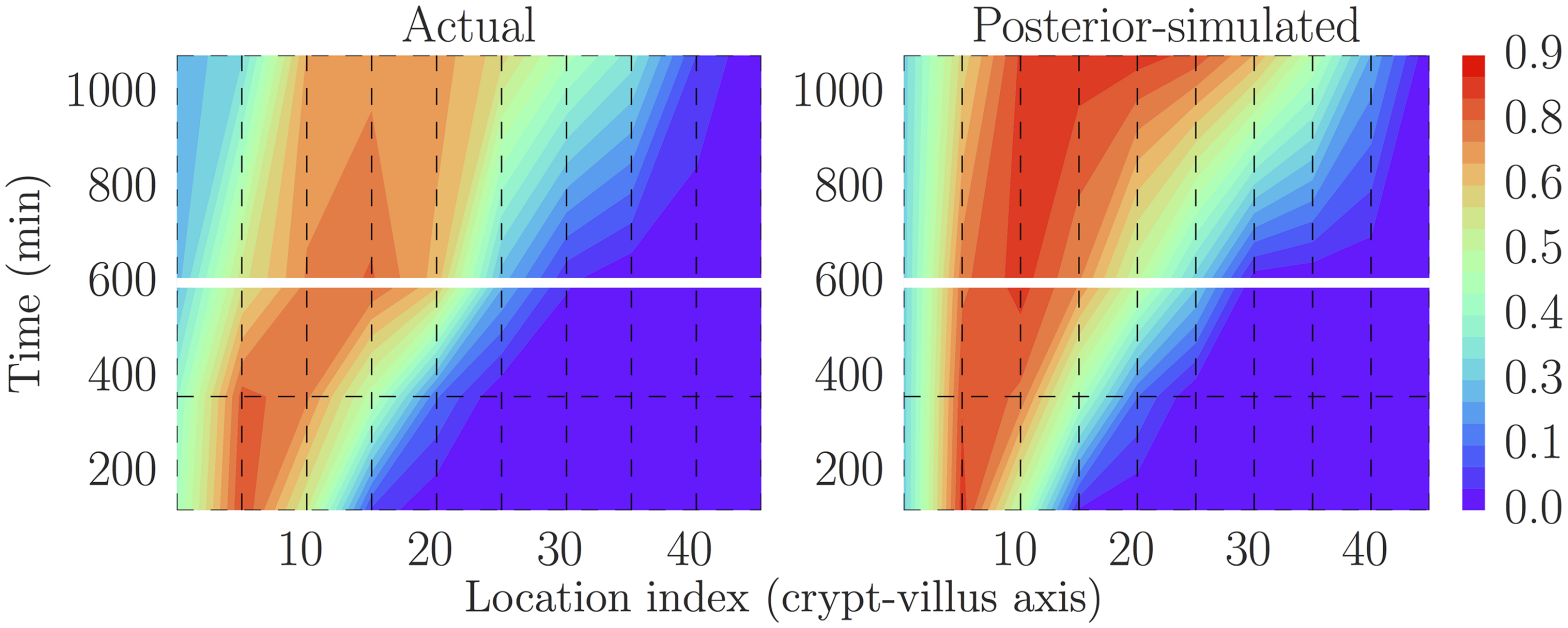
Actual (smoothed) data (left, black box) and one replication based on the model (right; plotting the latent/measurement-error-free process) as visualised in the characteristic plane. This has been discretised and interpolated between the dotted lines to facilitate fair but coarse-grained comparisons. The model structure implies that there should be lines of constant colour tracing out curves with slopes inversely proportional to the migration velocities at that point. The model again captures several of these key qualitative features, but fits less well for the out-of-sample (above the horizontal gap at 600 min/10 h) data. There is little variability in the latent model process and so only one replication is shown.

Figs 6 and 7 illustrate two additional ways of visualising replicated datasets. The former visualises the label profile along the crypt-villus axis at the future unfitted/out-of-sample time 1080 min (18 h), while the latter visualises both fitted (120 min/2 h, 360 min/6 h and 600 min/10 h) and unfitted/out-of-sample (1080 min/18 h) predictions plotted in the characteristic plane (*t*, *x*) in which the slopes along lines of constant colour should be inversely proportional to the migration velocities at that point, due to the (hyperbolic) nature of our ‘colour’ equation (see e.g. [59]). We have interpolated between the dotted grid lines. These figures, in combination with Fig 5, indicate that the model is capable of reliably reproducing the data to which it was fitted, as well as predicting key features of unfitted datasets such as the rate of movement of the front. On the other hand, there is clearly a greater misfit with the predicted rather than fitted data. In order to locate the possible source of misfit we considered various model residuals and error terms—see ‘Locating model misfit’ below.

### Predictive checks under blocked proliferation conditions

**Fig 8:**
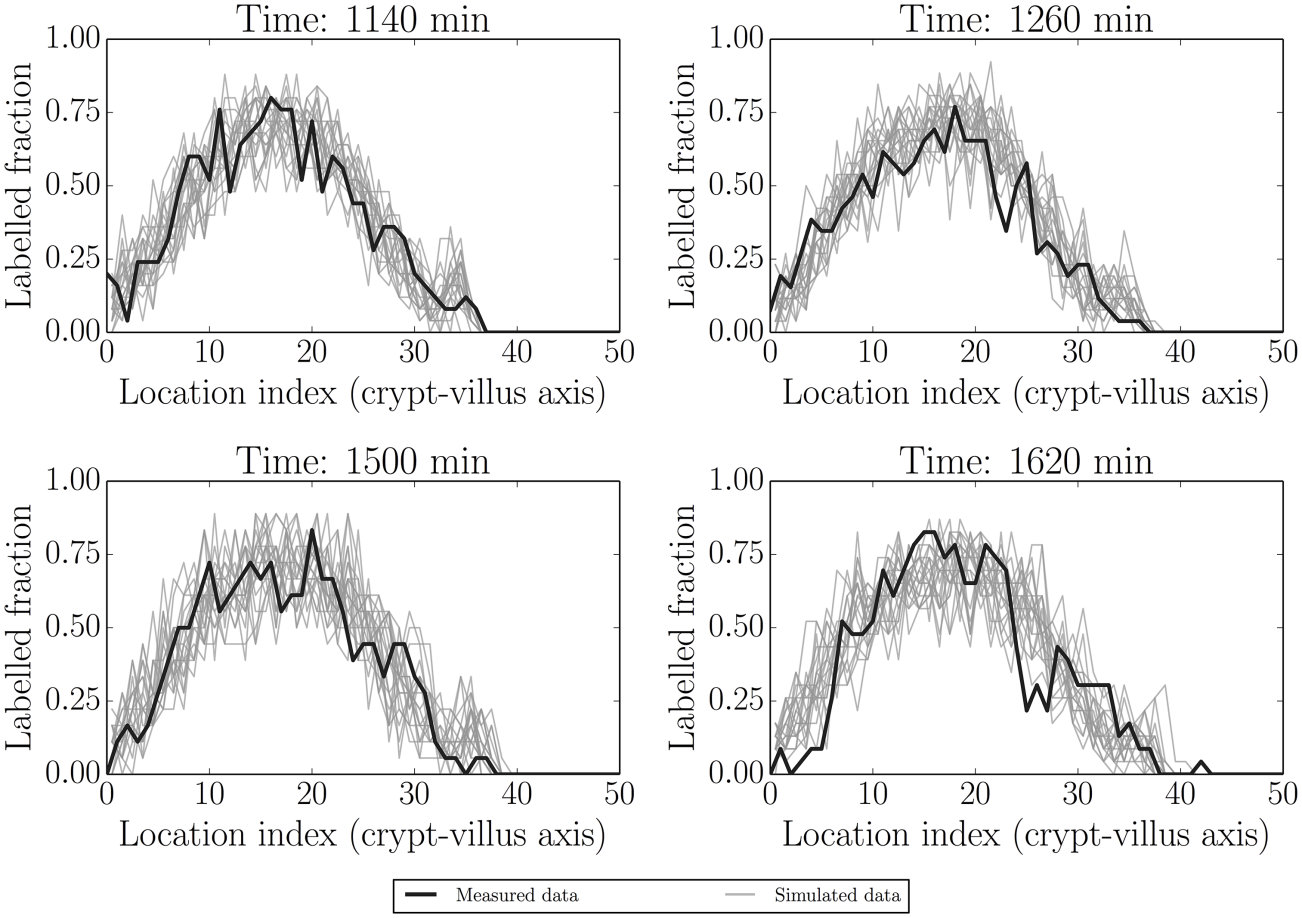
Simulated realisations from posterior predictive distributions (grey) for label data at 1140 min (initial condition), 1500 min (fitted time) and at two out-of-sample/unfitted times (1260 and 1620 min). The posterior distributions appear to adequately capture the actual label data (black).

Here data from 1140 min (19 h; post IdU labelling) were used as the initial conditions and 1500 min (25 h) used for fitting. 1260 min (21 h) and 1620 min (27 h) were used as out-of-sample (non-fitted) comparisons. Fig 8 is analogous to Fig 5 in the healthy case. In general all of the features up to 1620 min (27 h) in Fig 8, and for both fitted and predicted times, are reasonably well captured. The fit at 1620 min is generally good, but perhaps worse than the other cases. This could be due to errors in longer-time predictions and to the beginning of proliferation recovery: we explore these alternatives in what follows.

### Predictive checks under recovering proliferation conditions

As discussed above, Ara-C is metabolised between 10–12 h post-treatment. The two times considered here, 1620 min and 2520 min, correspond to 10 h and 25 h post Ara-C treatment, respectively, i.e to the end of the effect and after the resumption of proliferation.

**Fig 9:**
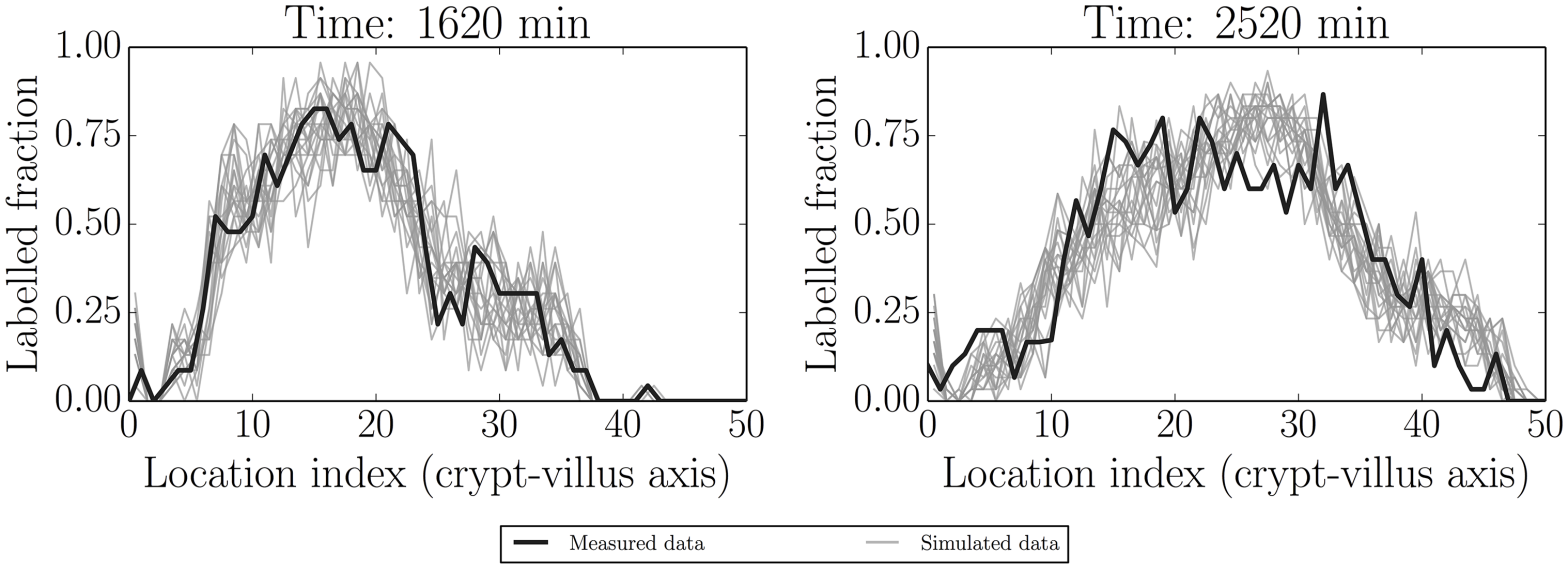
Simulated realisations from posterior predictive distributions (grey) for label data at 1620 min (initial condition) and 2520 min (fitted). These indicate that proliferation has resumed, consistent with the time taken to metabolise Ara-C—see the main text for more detail.

As can be seen in Fig 9, and as expected, the label has resumed movement in concert with the resumption in proliferation. The model appears to fit reasonably well.

### Locating model misfit

While the zeroth-order model behaves essentially as desired under experimental perturbation, and is likely capturing the essential features of interest, we observed some minor model misfit. We used posterior predictive checks to unpick the contributions of the various model parts and determine the source(s) of misfit. This in turn motivated potential model improvements. These checks were carried out under baseline (healthy) conditions as we were more confident of the experimental results under this scenario, but they can equally be carried out for the other datasets. Note, however, that time-varying effects are not expected to be as relevant under conditions of inhibited proliferation.

**Fig 10:**
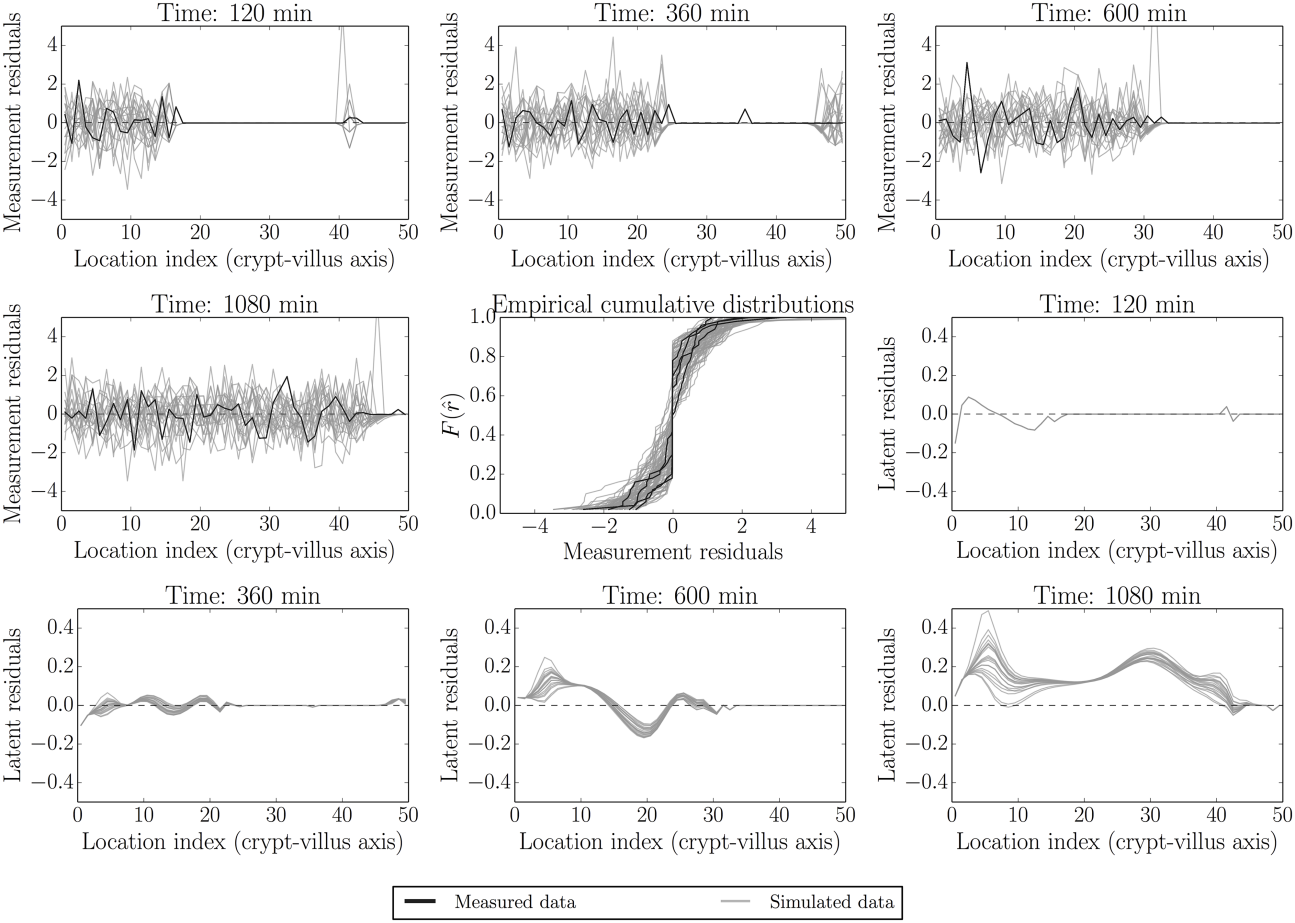
Model and data residual components. Panels 1–4, moving left-to-right and top-to-bottom, shows measurement error as determined by subtracting a smoothed spline from the observed data (dark line) and comparing this to the results obtained by subtracting the process model for fitted—120, 360 and 600 mins—and unfitted/out-of-sample—1080 min—times from the realised data (grey). These measurement error distributions are also visualised in terms of the corresponding cumulative distributions in the middle panel (panel 5, following as above. Black—actual data, grey—model simulations). Panels 6–9 show the differences between realisations of the underlying process model and the smoothed spline fitted to the data. As can be seen across panels, the measurement model appears approximately valid at all times, while the process model appears to have non-zero error for the 1080 min sample. This observation is discussed in the text.

Fig 10 shows the following checks: measurement error as determined by subtracting a smoothed spline from the observed data (dark line) and comparing these to the results obtained by subtracting the process model from the simulated data (panels 1–4, moving left-to-right and top-to-bottom, showing fitted—120 min/2 h, 360 min/6 h and 600 min/10 h—and unfitted/out-of-sample—1080 min/18 h—times). This presentation follows the noise-checking approach in [60], as well as the general recommendations given in [20, 58]. Reliable interpretation of these as ‘true’ measurement residuals depends on the validity of the normal approximation Eq 7 since these expressions are not directly interpretable in terms of the discrete binomial model (see e.g. [20, 58]). These are also visualised in terms of the corresponding cumulative distributions in the middle panel (panel 5, following as above). Panels 6–9 show the differences between the underlying process model and the smoothed spline fitted to the data. As can be seen, the measurement model appears approximately valid at all times, while the process model appears to have non-zero error for the 1080 min sample. We consider this in more detail next.

### Possible model improvement and robustness—Higher-order spatial effects

As discussed in the process model section above, the presence of cellular structure in the epithelial tissue means that higher-order spatial effects could be present. One way of deciding whether these are important is to consider the extent to which these may account for the minor misfit identified above, as opposed to other factors such as time-varying proliferation rates. To do this we considered both uniform percentage reductions of the original parameter estimates (approximating time-varying rates) and the inclusion of higher-order spatial terms.

**Fig 11:**
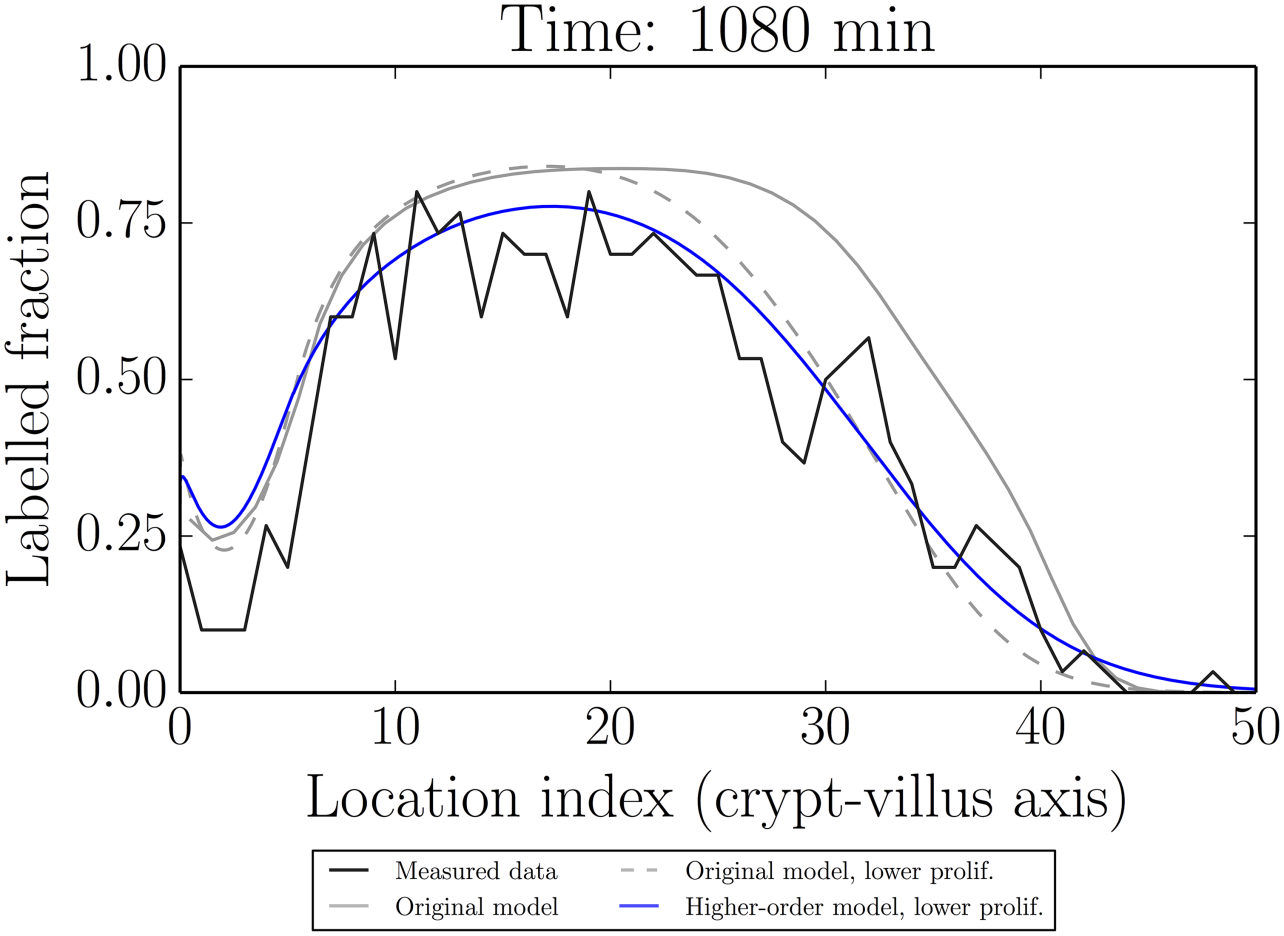
Comparison of the modified process model which includes higher-order spatial terms (blue) to the original model (grey, dashed), both at lowered proliferation rates (decreased 20%), which is required for a better fit to the data. The original model at the original fitted proliferation rates is also shown (grey, solid). Although the model with higher-order spatial terms gives a better qualitative fit to the data for the same proliferation rates, it is clear that the dominant cause of misfit is better attributed to (time) varying proliferation rates (in the context of the present set of models).

Fig 11 gives an idea of the qualitative differences induced by including the higher-order spatial terms and those that could be induced by time-varying proliferation rates. This figure is based on the (healthy) 1080 min (18 h) data in which we found some indication of a process model error.

We see that while the higher-order model appears to give a slightly better qualitative fit to the data, both the higher-order and lower-order models require similar reductions of the parameter values to quantitatively improve the fit to our out-of-sample data. The reduced parameter values shown in Fig 11 correspond to a reduction of 20%, which was chosen visually as a reduction accounting for the bulk of the misfit.

Thus the key (yet relatively small) difference between the model and out-of-sample data is likely due to an effect other than finite-cell sizes; in this case it is likely due to time-variation in parameter values due to circadian rhythms (we have assumed steady-state parameter values). Other potential factors include label dilution or an unmodelled mixing phenomenon in the full two-dimensional case. We note however that these effects are small and appear to be important primarily for predicting much further ahead in time than the fitted data and the steady-state parameter assumption is likely valid for reasonable time intervals. This means that the more easily interpretable original model may be sufficient for many purposes.

## Discussion

Understanding the intricate dynamics of the intestinal epithelium requires an interdisciplinary approach that integrates experimental measurements, mathematical and computational modelling, and statistical quantification of uncertainties. While a diverse range of mathematical models have been proposed for epithelial cell and tissue dynamics (reviewed in [51, 52, 61–63]), from compartment models to individual-based models to continuum models, we lack consistent and reproducible approaches for comparing models representing conjectured biological mechanisms both to each other and to experimental data (for an overview, see our review [44]). These shortcomings may explain why questions such as the connection between proliferation and migration and its variation under experimental perturbations remain open [8–14].

The aim of the present work was to acknowledge and confront these difficulties explicitly, and to present some initial constructive steps towards establishing such a framework. To do this we carried out new experiments (described more fully in a companion paper [18]) aimed at determining how cell proliferation rates, tissue growth and cell migration rates are related in the intestinal epithelium under healthy, damaged (Ara-C treated) and recovering conditions. We performed BrdU/IdU cell-labelling experiments under these respective conditions. We then developed a probabilistic, hierarchical (conditional) model to process and interpret these data.

Our hierarchical model provides a best-practice approach for describing and understanding the uncertainties that could lead to unreliable mechanistic conclusions—uncertainties in experimental measurement and treatment, difficult-to-compare mathematical models of underlying mechanisms, and unknown or unobserved parameters. Our approach was influenced by recognising the benefits that the hierarchical Bayesian approach has demonstrated in applications across a number of different disciplines (e.g. in environmental and geophysical science as in [64, 65]; ecological modelling as in [66, 67]; and in Bayesian statistical modelling and inverse problems more generally as in [20–24, 68]). We also note that a hierarchical approach can have significant benefits outside the Bayesian framework (see for example the ‘extended likelihood’ approach described in [69–71]).

The hierarchical approach provides a framework, not only for combining disparate sources of uncertainty, but also for facilitating modelling derivations and relating discrete and continuous models. Though the resulting measurement, process and parameter models can be (or have been) derived by other means, as far as we are aware this particular perspective has not been systematically utilised in the manner considered here.

We also note the connection between the choice of a measurement model as required here (and/or process model error, and following e.g. [21–24, 64, 72]), and the development of approximate sampling and parameter fitting procedures, which are particularly useful for analytically difficult models. A key concern of the latter is the appropriate choice of summary statistics for constructing a ‘synthetic likelihood’ [73] or similarly-modified posterior target for Approximate Bayesian Computation (ABC) [74–76]. This choice determines (implicitly or explicitly) in which ways a given model or set of models can be considered an ‘adequate’ representation of the data, which features are considered to be reproducible and what the associated ‘noise’ structure should be ([77] presents an alternative approach to characterising data features and model adequacy). These issues are crucial in deciding how to model the complexity of epithelial cell and tissue dynamics.

An important next step, as described above, would be to consider other process model types and to evaluate and compare them under carefully modelled experimental conditions. Extensions incorporating other mechanical and/or cellular-level information (e.g. [11, 12]) into process models would provide a natural next step. Importantly, due to the separation between measurement and process model components, these more complex process models could be incorporated into our present framework simply by replacing our process model component with a new model, while retaining the same measurement model. Of course additional parameters would require additional prior assumptions, and if additional data features were of interest then these would need to be incorporated into a modified measurement model. The benefit of a hierarchical model is that it offers an explicit guide as to where such modifications should be incorporated.

In summary, the main results established using the above approach were

- An adequate description of intestinal epithelial dynamics is achievable using a model based on proliferation-driven growth alone;
- This model is consistent with healthy, proliferation-inhibited (Ara-C treated) and recovering conditions;
- The measurement and process model errors can be reasonably distinguished and checked separately;
- Checking indicates that much of the natural variability is attributable to the data collection process and this process can be modelled in a simple manner
- Possible model errors can also be identified and proposed explanations incorporated and tested within our model, and, thus, the proper interpretation of experimental procedures is aided by using an explicit mathematical model and its predictive simulations
- Including finite-cell-size effects gives a slightly better qualitative fit to experimental data, but the dominant sources of the long-time misfits are likely due to some other factor such as (relatively slowly) time-varying proliferation rates (e.g. due to circadian rhythms) or label dilution.

## Supporting information

S1 Supplementary information **Fig A**. **Posterior for proliferation rates under baseline, healthy conditions**. The upper diagonal represents the marginal distributions for each proliferation rate when averaging over all other profileration rates. The plots below the diagonal show bivariate marginal distributions illustrating pairwise associations after averaging over all other profileration rates. These visualisations are a way of understanding the full joint posterior distribution which is five-dimensional in full generality. **Fig B**. **Posterior for proliferation rates under Ara-C treatment**. The upper diagonal represents the marginal distributions for each proliferation rate when averaging over all other profileration rates. The plots below the diagonal show bivariate marginal distributions illustrating pairwise associations after averaging over all other profileration rates. These visualisations are a way of understanding the full joint posterior distribution which is five-dimensional in full generality. **Fig C**. **Posterior for proliferation rates when recovering from Ara-C treatment**. The upper diagonal represents the marginal distributions for each proliferation rate when averaging over all other profileration rates. The plots below the diagonal show bivariate marginal distributions illustrating pairwise associations after averaging over all other profileration rates. These visualisations are a way of understanding the full joint posterior distribution which is five-dimensional in full generality. **Fig D**. **Typical section obtained during the experimental procedures described in the main manuscript**. These are also described more fully in the companion paper [18].
(PDF)
