# Supplementary material for: A hierarchical Bayesian model for understanding the spatiotemporal dynamics of the intestinal epithelium
Source: PLoS Comput Biol. 2017 Jul 28;13(7):e1005688. doi: 10.1371/journal.pcbi.1005688 (PMC5550005; doi:10.1371/journal.pcbi.1005688)
Supplement: S1 Supplementary information — Fig A. Posterior for proliferation rates under baseline, healthy conditions. The upper diagonal represents the marginal distributions for each proliferation rate when averaging over all other profileration rates. The plots below the diagonal show bivariate marginal distributions illustrating pairwise associations after averaging over all other profileration rates. These visualisations are a way of understanding the full joint posterior distribution which is five-dimensional in full generality. Fig B. Posterior for proliferation rates under Ara-C treatment. The upper diagonal represents the marginal distributions for each proliferation rate when averaging over all other profileration rates. The plots below the diagonal show bivariate marginal distributions illustrating pairwise associations after averaging over all other profileration rates. These visualisations are a way of understanding the full joint posterior distribution which is five-dimensional in full generality. Fig C. Posterior for proliferation rates when recovering from Ara-C treatment. The upper diagonal represents the marginal distributions for each proliferation rate when averaging over all other profileration rates. The plots below the diagonal show bivariate marginal distributions illustrating pairwise associations after averaging over all other profileration rates. These visualisations are a way of understanding the full joint posterior distribution which is five-dimensional in full generality. Fig D. Typical section obtained during the experimental procedures described in the main manuscript. These are also described more fully in the companion paper [18]. (PDF) [file pcbi.1005688.s001.pdf]

Supplementary information

Availability

All manuscript, code and data files can be found at the following github page:

https://github.com/omaclaren/open-manuscripts/tree/master/hierarchical-framework-intestinal-epithelium

Derivation of 'zeroth-order' continuous model

To derive the continuous approximation we first defined the position x as a continuous coordinate passing through the discrete cell indices. For example $x = 0$ denoted the coordinate of the cell labelled '0' (base of the crypt), while $x = 0.5$ was the location halfway between the cell labelled '0' and that labelled 1'. Sample locations consisting of space-time pairs were denoted by $s = (x_s, t_s)$. Then, for sample locations (i, t) corresponding to cell indices and arbitrary times, we matched the discrete model and continuous model using

$$p(l_i(t) = 1|L(i,t)) = L(i,t) \tag{1}$$

i.e. $L(i, t)$ served as the parameter for a single measurement modelled as a Bernoulli trial at that sample location (as in the above Measurement model section).

Next, the discrete dynamics of $p(l_i(t) = 1)$ were 'transferred' to the continuous $L(x, t)$ dynamics. In particular, since $L(x, t)$ was taken to be a smooth function, we made the correspondence

$$\begin{gathered} p(l_{i-1}(t) = 1|L(i-1,t)) = L(i-1,t) \\ \equiv \\ p(l_{i-1}(t) = 1|L(i,t), L_x(i,t), ..., \Delta x) = L(i,t) - \Delta x \frac{\partial L(i,t)}{\partial x} + \frac{\Delta x^2}{2}\frac{\partial^2 L(i,t)}{\partial x^2} - ... \end{gathered} \tag{2}$$

where $\Delta x = i - (i - 1) = 1$ was the normalised cell length and we also conditioned on knowledge of the spatial derivatives at i, $L_x(i, t) = \frac{\partial L(i,t)}{\partial x}$ etc. The continuous spatial field effectively interpolated between - i.e. *internal* to - points of the discrete grid, making use of local derivative information. Substituting the above Taylor series, and similar expressions, into the discrete Markov equation led to

$$\frac{\partial L(i,t)}{\partial t} + v(i)\frac{\partial L(i,t)}{\partial x} = \frac{1}{2}\left(\Delta x v(i)\frac{\partial^2 L(i,t)}{\partial x^2} - \Delta t\frac{\partial^2 L(i,t)}{\partial t^2}\right) + ... \quad (3)$$

where, for completeness, we also retained higher order terms in Δt for the continuous model. We similarly assumed the existence of smooth functions $k(x,t)$ and $v(x,t)$ that satisfied the discrete relations

$$v(i,t) = \sum_{j=0}^{i-1} k_j \Delta x = \int_0^i k(x,t)dx + v(0). \quad (4)$$

Furthermore, we assumed $k(x,t) = k(x)$, $v(x,t) = v(x)$ and $v(0) = 0$ in what follows. This assumption is discussed further in the Results section.

We obtained 'closure' for the continuous model by keeping only the lowest order terms in both time and space, and further asserting that the equation structure obtained held *for all continuous* x and not just discrete i (this could also be motivated by an assumption of grid translation invariance). This leads to the advection equation

$$\frac{\partial L(x,t)}{\partial t} + v(x)\frac{\partial L(x,t)}{\partial x} = 0 \quad (5)$$

with

$$v(x) = \int_0^x k(x')dx'.$$

When we incorporated cell death, with discrete rates d_i, this led to the same equations with k replaced by $k - d$, where $d(x,t)$ was defined similarly to $k(x,t)$. Hence we interpreted k in the above as the net cell production rate (which hence could be negative).

Supplementary visualisations of posterior distributions

In Fig A, Fig B and Fig C, respectively, we present alternative visualisations of the posterior distributions for proliferation rates under healthy, Ara-C-treated and recovering conditions. These are alternative visualisations of the data presented in Figs 3-5 in the main manuscript. These plots were produced using the package 'corner.py' described in [1].

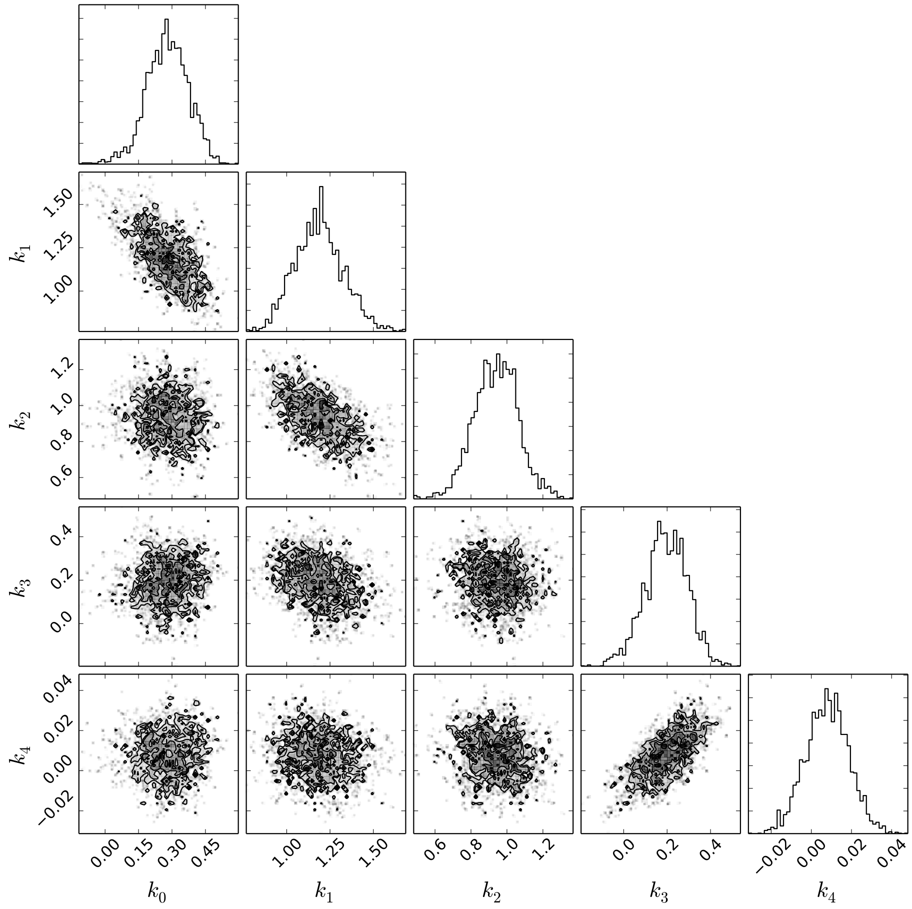

Fig A. Posterior for proliferation rates under baseline, healthy conditions. The upper diagonal represents the marginal distributions for each proliferation rate when averaging over all other profileration rates. The plots below the diagonal show bivariate marginal distributions illustrating pairwise associations after averaging over all other profileration rates. These visualisations are a way of understanding the full joint posterior distribution which is five-dimensional in full generality.

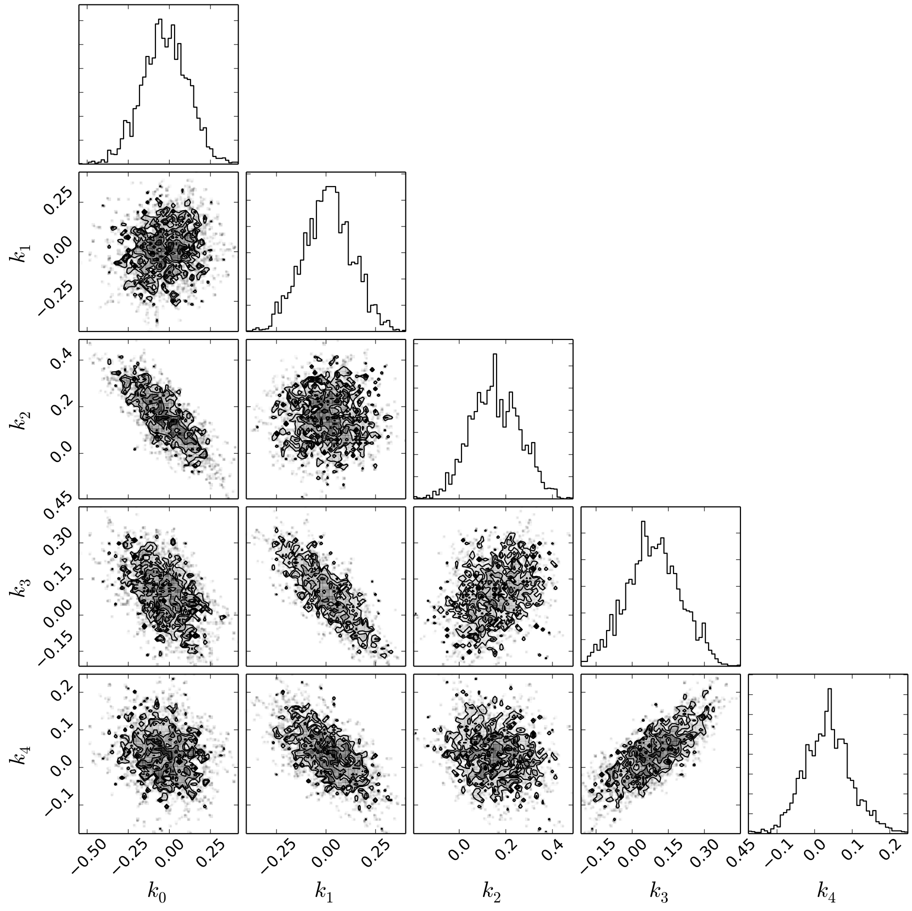

Fig B. Posterior for proliferation rates under Ara-C treatment. The upper diagonal represents the marginal distributions for each proliferation rate when averaging over all other profileration rates. The plots below the diagonal show bivariate marginal distributions illustrating pairwise associations after averaging over all other profileration rates. These visualisations are a way of understanding the full joint posterior distribution which is five-dimensional in full generality.

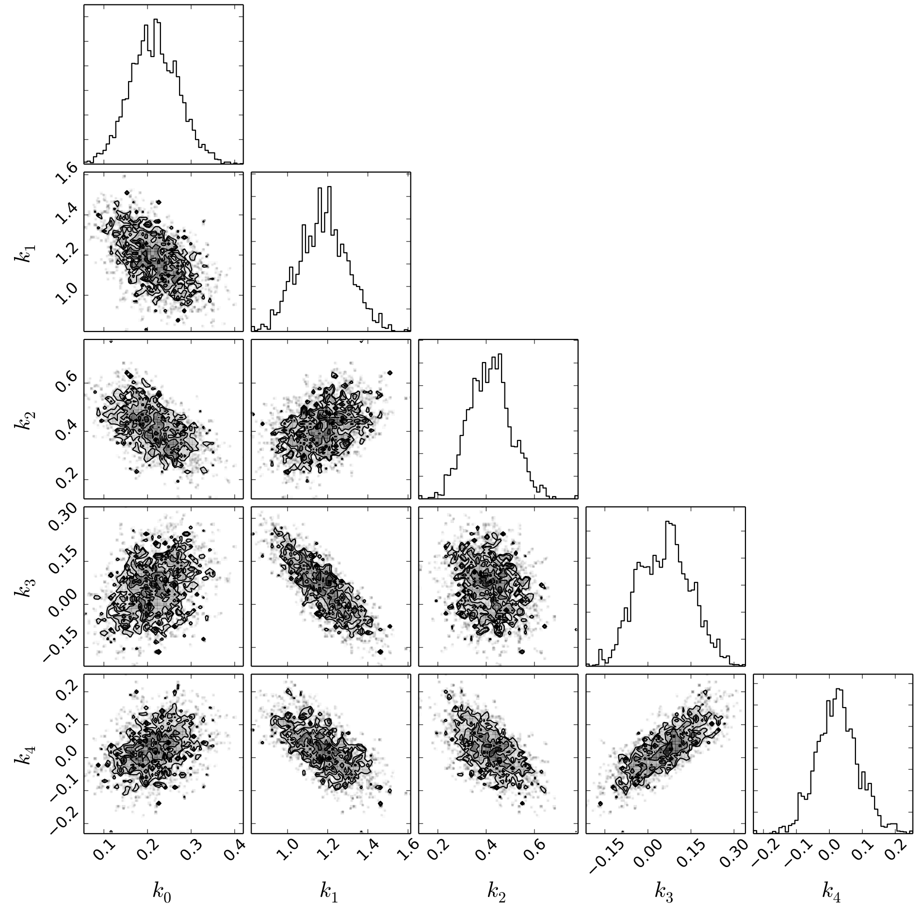

Fig C. Posterior for proliferation rates when recovering from Ara-C treatment. The upper diagonal represents the marginal distributions for each proliferation rate when averaging over all other profileration rates. The plots below the diagonal show bivariate marginal distributions illustrating pairwise associations after averaging over all other profileration rates. These visualisations are a way of understanding the full joint posterior distribution which is five-dimensional in full generality.

Typical sample from intestinal epithelium

The companion paper [2] contains full details of the experimental procedures. In Fig D below we reproduce, for reference, a typical section obtained from an intestinal in these experiments.

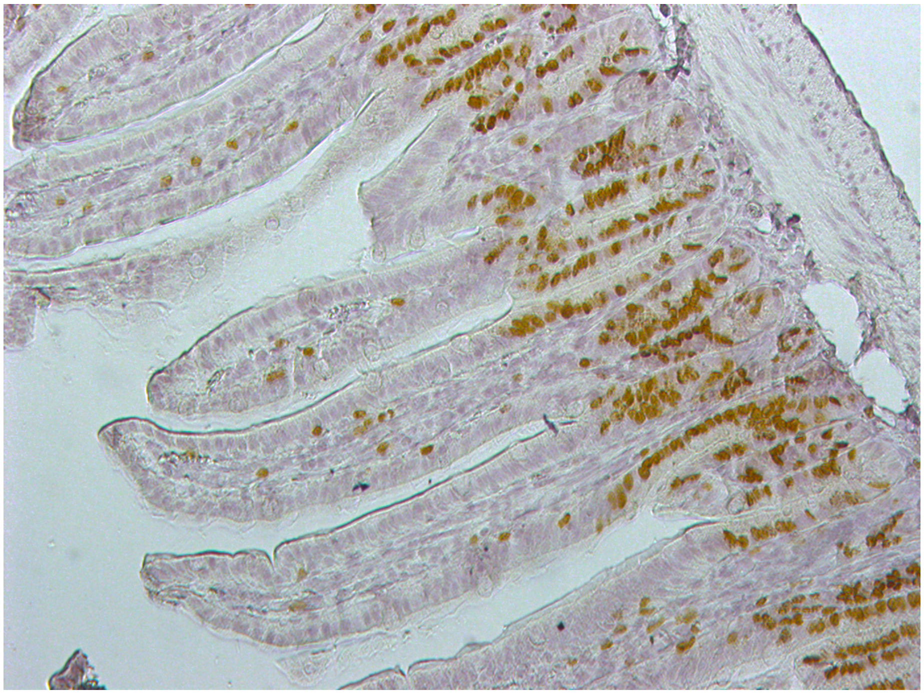

Fig D. Typical section obtained during the experimental procedures described in the main manuscript. These are also detailed more fully in the companion paper [2].

Interpretation of statistical evidence

We have described above how mechanistic or causal assumptions relate to assumptions of structural invariance under different scenarios. In order to interpret the results that follow, however, we also required an interpretation of the 'statistical evidence' that a set of measurements provided about parameter values within a fixed model structure. This proved a surprisingly controversial topic and we encountered continuing debate about fundamental principles and definitions of statistical evidence [3–7].

Following our conditional modelling approach, we decided to adopt the simple - yet quite generally applicable - principle of evidence based on conditional probability: if we observe b and $p(a|b) > p(a)$ then we have evidence for a. A

'gold-standard' theory of statistical evidence starting from this premise has been developed and defended recently by Evans in a series of papers (summarised in [6]). Besides simplicity, a nice feature of this approach, that we used below, is that it can be applied both to prior and posterior predictive distribution comparisons such as $p(\mathbf{y}|\mathbf{y_0}) \overset{?}{>} p(\mathbf{y})$, as well as to prior and posterior parameter distribution comparisons such as $p(\mathbf{k}|\mathbf{y_0}) \overset{?}{>} p(\mathbf{k})$. This approach is not without criticism, however (again, see [3–7] for an entry point to the ongoing debates).

Another notable feature of the interpretation of statistical evidence that we adopted below is that we emphasised the visual comparison of various prior and posterior distributions, rather than adopting arbitrary numerical standards ([8] advocates a similar 'movie strategy' for the interpretation of statistical evidence and inference procedures, [9–12] similarly emphasise the benefits of graphical visualisation methods in statistics).

1. Foreman-Mackey D (2016) Corner.py: Scatterplot matrices in python. The Journal of Open Source Software 1:1–2

2. Parker A, Maclaren OJ, Fletcher AG, Muraro D, Kreuzaler PA, Byrne HM, Maini PK, Watson AJM, Pin C (2016) Cell proliferation within small intestinal crypts is the principal driving force for cell migration on villi. The FASEB Journal; published ahead of print October 20, 2016, doi:10.1096/fj.201601002

3. Royall R (1997) Statistical evidence: A likelihood paradigm. CRC Press

4. Mayo DG (2014) On the birnbaum argument for the strong likelihood principle. Stat Sci 29:227–239

5. Evans M (2014) Discussion of "on the birnbaum argument for the strong likelihood principle". Stat Sci 29:242–246

6. Evans M (2015) Measuring statistical evidence using relative belief. CRC Press

7. Taper ML, Ponciano JM (2016) Evidential statistics as a statistical modern synthesis to support 21st century science. Popul Ecol 58:9–29

8. Tarantola A (2005) Inverse problem theory and methods for model parameter estimation. SIAM

9. Gelman A (2004) Exploratory data analysis for complex models. J Comput Graph Stat 13:755–779

10. Gelman A, Carlin JB, Stern HS, Dunson DB, Vehtari A, Rubin DB (2013) Bayesian data analysis, third edition. Taylor & Francis

11. Gelman A, Shalizi CR (2013) Philosophy and the practice of Bayesian statistics. Br J Math Stat Psychol 66:8–38

12. Davies PL (2014) Data analysis and approximate models: Model choice,

Location-Scale, analysis of variance, nonparametric regression and image analysis. CRC Press
